# The enigmatic mitochondrial genome of *Rhabdopleura compacta *(Pterobranchia) reveals insights into selection of an efficient tRNA system and supports monophyly of Ambulacraria

**DOI:** 10.1186/1471-2148-11-134

**Published:** 2011-05-20

**Authors:** Marleen Perseke, Joerg Hetmank, Matthias Bernt, Peter F Stadler, Martin Schlegel, Detlef Bernhard

**Affiliations:** 1 Molecular Evolution and Animal Systematics, University of Leipzig, Talstr. 33, 04103 Leipzig, Germany; 2 Laboratory of Marine Biology, South China Sea Institute of Oceanology, Chinese Academy of Science, 164 West Xingang Road, 510301 Guangzhou, PR China; 3 Parallel Computing and Complex Systems Group, University of Leipzig, Johannisgasse, 26, 04103 Leipzig, Germany; 4 Bioinformatics Group, Dept. of Computer Science, University of Leipzig, Härtelstr, 16-18, 04107 Leipzig, Germany; 5 Interdisciplinary Center for Bioinformatics, University of Leipzig, Härtelstr, 16-18, 04107 Leipzig, Germany; 6 Max Planck Institute for Mathematics in the Sciences, Inselstrasse 22, 04103 Leipzig, Germany; 7 Fraunhofer Institut für Zelltherapie und Immunologie, IZI, Perlickstrasse 1, 04103 Leipzig, Germany; 8 Center for non-coding RNA in Technology and Health, University of Copenhagen, Grønnegårdsvej 3, 1870 Frederiksberg C, Denmark; 9 Department of Theoretical Chemistry, University of Vienna, Währingerstrasse 17, 1090 Wien, Austria; 10 Santa Fe Institute, 1399 Hyde Park Rd, Santa Fe, NM 87501, USA

**Keywords:** Hemichordata, Pterobranchia, deuterostome evolution, codon reassignment, codon-anticodon adaptation

## Abstract

**Background:**

The Hemichordata comprises solitary-living Enteropneusta and colonial-living Pterobranchia, sharing morphological features with both Chordata and Echinodermata. Despite their key role for understanding deuterostome evolution, hemichordate phylogeny is controversial and only few molecular data are available for phylogenetic analysis. Furthermore, mitochondrial sequences are completely lacking for pterobranchs. Therefore, we determined and analyzed the complete mitochondrial genome of the pterobranch *Rhabdopleura compacta *to elucidate deuterostome evolution. Thereby, we also gained important insights in mitochondrial tRNA evolution.

**Results:**

The mitochondrial DNA of *Rhabdopleura compacta *corresponds in size and gene content to typical mitochondrial genomes of metazoans, but shows the strongest known strand-specific mutational bias in the nucleotide composition among deuterostomes with a very GT-rich main-coding strand. The order of the protein-coding genes in *R. compacta *is similar to that of the deuterostome ground pattern. However, the protein-coding genes have been highly affected by a strand-specific mutational pressure showing unusual codon frequency and amino acid composition. This composition caused extremely long branches in phylogenetic analyses. The unusual codon frequency points to a selection pressure on the tRNA translation system to codon-anticodon sequences of highest versatility instead of showing adaptations in anticodon sequences to the most frequent codons. Furthermore, an assignment of the codon AGG to Lysine has been detected in the mitochondrial genome of *R. compacta*, which is otherwise observed only in the mitogenomes of some arthropods. The genomes of these arthropods do not have such a strong strand-specific bias as found in *R. compacta *but possess an identical mutation in the anticodon sequence of the tRNA_Lys_.

**Conclusion:**

A strong reversed asymmetrical mutational constraint in the mitochondrial genome of *Rhabdopleura compacta *may have arisen by an inversion of the replication direction and adaptation to this bias in the protein sequences leading to an enigmatic mitochondrial genome. Although, phylogenetic analyses of protein coding sequences are hampered, features of the tRNA system of *R. compacta *support the monophyly of Ambulacraria. The identical reassignment of AGG to Lysine in two distinct groups may have occurred by convergent evolution in the anticodon sequence of the tRNA_Lys_.

## Background

Pterobranchia are a small, specialised group of marine suspension-feeding animals. Despite rich fossil record, only some 20 extant species have been described, which are subdivided into three genera, *Rhabdopleura, Cephalodiscus *and *Atubaria *[1]. One questionable species from the genus *Atubaria *(*Atubaria heterolopha*) is described, which lives solitary [2]. Species of *Rhabdopleura *and *Cephalodiscus *live in secreted tubes in colonies [1]. Although Pterobranchia comprise only few extant species, they represent an important deuterostome lineage. Usually they are grouped to the solitary-living Enteropneusta, forming together the Hemichordata (e.g. [3]). Because the Hemichordata unite features of the remaining two major deuterostome subgroups, Chordata and Echinodermata, the phylogenetic position of Pterobranchia is crucial for understanding the evolution of chordate and deuterostome body plans.

Despite their evolutionary importance, only ribosomal sequence data were analysed for phylogenetic reconstruction of the pterobranchs [4-6]. These analyses support the monophyly of Hemichordata and a close relationship with Echinodermata, forming the Ambulacraria [7]. However, results within this group are conflicting. The *28S rDNA *sequence analyses suggest reciprocal monophyly of Enteropneusta and Pterobranchia while the analyses of the *18S rDNA *sequences support a branching of Pterobranchia within Enteropneusta.

A frequently used molecular marker for reconstruction of the phylogenetic relationships is the complete mitochondrial genome (e.g. [8-11]). The typical metazoan mitochondrial genome is a circular DNA molecule ranging from 15 to 20 kb in size and comprises a more or less conserved gene content of 13 protein-coding genes, two ribosomal RNA (rRNA) genes and 22 transfer RNA (tRNA) genes [12]. The complete genomes allow phylogenetic analyses of both sequences and gene order. The complete mitochondrial genomes are well characterised from Vertebrata (e.g. [12]), Cephalochordata [9], Tunicata (e.g. [13]), and the five recent echinoderm subgroups (e.g. [11]). The mitochondrial genome was also determined for the enigmatic worm-like *Xenoturbella bocki *supporting a close relationship to Deuterostomia [10,14]. With the exception of the highly derived Tunicata, mitochondrial genomes of Deuterostomia are similar in architecture [8]. However, variability has been found in the genetic code caused by different assignments of four codons (AGA, AGG, AAA, and AUA). Since the metazoan mitochondrial translation system comprises a reduced tRNA gene set of usually 22 tRNA genes, each tRNA gene has to recognize different codons (usually two or four codons), which often differ only in the 3^rd ^codon site. The influence of the 3^rd ^codon site on the translation efficiency is still under discussion [15-22]. The "theory of anticodon sequences with highest versatility" postulates that the anticodon sequence of a tRNA gene pairs equally effectively with all recognized codons [15,16,18]. Alternatively, the "theory of codon-anticodon adaptation" suggests that the anticodon sequence of a tRNA gene is adapted to the most frequent codon among all the recognized codons [19,20]. As most metazoan mitochondrial genomes show an AT-rich main-coding strand [23], the tRNAs preferentially have GNN anticodons for NNY codons, UNN for NNR codons, and also UNN for the four-fold degenerate codon families [19,20]. This is in congruence with both theories (see [17]). The strand-specific nucleotide bias may have arisen by the asymmetrical replication of the circular mitochondrial genome which exposes stretches of a DNA strand for a longer time as single-stranded than other parts, accumulating Adenine and Guanine nucleotides more faster [23-28]. However, some mitochondrial genomes possess an inverse strand-specific mutational pattern showing a GT-rich main-coding strand, e.g. *Branchiostomia *(Cephalochordata) and Crinoidea (Echinodermata), which is usually explained by an inversion of the replication direction of the mtDNA [23,29].

In contrast to these well characterized genomes, only three enteropneust genomes are known from Hemichordata. The two *Balanoglossus *genomes exhibit similarities to Vertebrata and Echinodermata [30]. The analyses of the genetic code suggest assignments as found in Echinodermata, although two important codons are missing (AAA and AAG) [31]. The genome of the enteropneust *Saccoglossus kowalevskii *was not described in detail, but the published annotated sequence shows a derived gene order and a different assignment of the codon AAA compared to the echinoderm genetic code (AAA ≠ Asn; NC_007438). However, this assignment in the genome of *S. kowalevskii *(AAA = Lys) also occurs in other invertebrate mtDNAs.

We determined the first mitochondrial genome of a pterobranch, *Rhabdopleura compacta*, and compared the genome architecture and the genetic code to other deuterostome and metazoan genomes. The genome of *R. compacta *shows an unusual strand-specific bias, which strongly affected the protein sequences, and suggests an inversion of the replication direction. This precluded the phylogenetic reconstruction of Deuterostomia including Pterobranchia by protein sequence analyses. The order of protein-coding genes of *R. compacta *is similar to the hypothetical ancestral arrangement of deuterostomes but is also not informative for phylogenetic reconstruction within the Deuterostomia. The codon-anticodon distribution and the codon assignments in the pterobranch genome reject an adaptation of anticodon sequences to the most frequent codons, but give strong support for selection to an efficient tRNA translation system with anticodon sequences showing highest versatility. Features of its tRNA system support the grouping of Ambulacraria (Echinodermata and Hemichordata).

## Results and Discussion

### General Features of the Mitochondrial Genome of *Rhabdopleura compacta*

**Figure 1:**
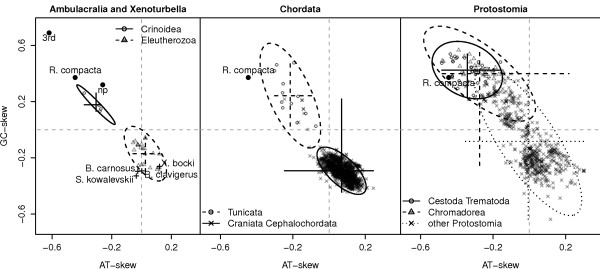
**Nucleotide skews for the main-coding strand of the pterobranch *Rhabdopleura compacta *compared to other bilaterian mitochondrial genomes**. Values of the nucleotide skews of *R. compacta *are marked by filled black circles (3rd: values for the 3rd codon position of all protein-coding genes; np: values for non- protein coding regions). The nucleotide skew values for other bilaterian mitochondrial genomes are marked by different symbols as indicated in each figure. The values of different phylogenetic groups are visualized by a cross giving the minimum and maximum of the nucleotide skews, and an ellipse with major axis in the direction of the eigenvector of the covariance matrix. Note the strong deviation of the nucleotide skews in *R. compacta*, similar to the mitochondrial genomes of the parasitic flatworms (Cestoda, Trematoda) and certain nematodes (Chromadorea).

The mitochondrial genome of *R. compacta *comprises a 15,814 bp long circular DNA molecule with a slightly higher AT-content (66%) than enteropneust genomes (52 - 60%). It shows one of the strongest strand-specific biases of metazoan genomes with high amounts of Thymine (48%) and Guanine (23%) but low amounts of Adenine (18%) and Cytosine (11%) on the main-coding strand causing strong negative AT- and positive GC-skews (Figure 1). The main-coding strand bears several oligoT-stretches with lengths of six (48 times), seven (38 times), eight (19 times) and nine nucleotides (10 times), while the longest stretch of 13 T's is found only once. The large amount of Thymine leads to a higher total AT-content, although the GT-content is also markedly increased in all *Rhabdopleura *mt genes compared to closely related taxa (Table 1).

**Table 1:** AT- and GT-content of mtDNA genes from *R. compacta *compared to mtDNAs of Enteropneusta.

		*Rhabdopleura compacta*	*Balanoglossus carnosus*	*Balanoglossus clavigerus*
**tRNA genes**	**AT-content % **(total)	**65.7 **(964)	**52.6 **(823)	**53.4 **(832)
	
	**GT-content % **(total)	**56.7 **(833)	**51.0 **(797)	**50.8 **(792)

**rRNA genes**	**AT-content % **(total)	**68.8 **(1502)	**55.2 **(1276)	**55.4 **(1283)
	
	**GT-content % **(total)	**64.3 **(1404)	**43.1 **(998)	**42.6 **(986)

**Protein-coding genes**	**AT-content % **(total)	**65.7 **(8012)	**50.5 **(5851)	**52.4 **(6209)
	
	**GT-content % **(total)	**73.4 **(8955)	**45.4 **(5260)	**45.2 **(5359)

**Figure 2:**
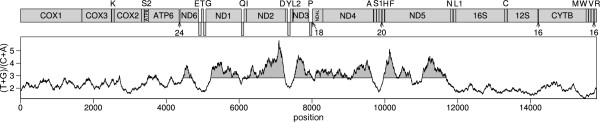
**Linear map of the mitochondrial genome of the pterobranch *Rhabdopleura compacta *with the (G + T)/(A + C) ratios at each nucleotide position computed for windows of size 300 (see Methods)**. The nucleotides were counted starting with *COX1 *and are shown on the bottom scale. Genes located above the middle line are transcribed from the heavy strand whereas those located below the middle line are transcribed from the light strand. UAS regions > 16 bp are indicated by arrows with the number showing their length. The values of a (G + T)/(A + C) ratio larger than slope (m = 2.824) of a fitted linear model are shaded.

The genome contains all genes typical for Metazoa, i.e., 13 protein-coding, two rRNA, and 22 tRNA genes (Figure 2). All protein-coding genes start with ATG or GTG and end with a complete stop-codon with the exception of *COX3 *and *ND4*. Both genes overlap with downstream tRNA genes suggesting an incomplete termination codon "T" as described in Ojala et al. [32]. The *ATP8 *protein is unusual: about 20 residuals of the C-terminus are missing. In contrast, additional parts within the protein sequences are detected within *ATP6 *(about 50 aa) and *ND3 *(about 28 aa). All protein-coding genes are located on the same strand and furthermore, the strand-specific nucleotide skews are predominantly found in protein-coding genes (Figure 1 and 2). Moreover, the oligoT-stretches are frequently located in protein-coding regions. The longest T-stretch is located within the coding-region of *COX3 *leading to four phenylalanine residues in series within an evolutionary conserved phenylalanine-rich domain.

The 22 tRNA genes have been identified based on the predicted cloverleaf secondary structure. The increased GT-content has also been observed in the rRNA and, to a lesser extent, in the tRNA genes (Table 1). The two rRNA genes have been detected by alignments. Their boundaries have been recovered only for the *12S rRNA*, whereas the *16S rRNA *has been determined by the flanking tRNA genes. The *12S rRNA *is 842 bp in size and similar to the *12S rRNA *of the enteropneusts (815 bp - 851 bp) showing 41 - 42% sequence identities. The *16S rRNA *(≤1,342 bp) is much shorter than the enteropneust sequences (1,498 bp - 1,504 bp) but exhibits 41 - 42% sequence identity with them. The exact ends of the *16S rRNA *is uncertain, the gene is possibly surrounded by short UAS (unassigned sequences).

Surprisingly, only 1% of the genome consists of UAS and there is no large UAS region at all. The longest UAS region spans 24 bp and is located between the *ATP6 *and *ND6 *genes (Figure 2).

### Strand-specific Nucleotide Biases

Almost all metazoan mitochondrial genomes present obvious strand-specific AT- and GC-skews (Figure 1) which is usually explained by the (asymmetrical) strand-displacement mechanisms inherent in the replication of mitogenomes [23]. Vertebrate genomes present pronounced AT- and GC-skews on the two strands having an AC-rich main-coding strand (Figure 1) (e.g. [25]). This strand-specific mutational bias is explained by the asymmetrical replication. Replication starts from two distinct replication initiation sites for the GT-rich (heavy) strand (oriH) and the AC-rich (light) strand (oriL) [24,25], respectively. While the replication of the genome starts on oriH, the replication of the light strand starts later on oriL, when the replication fork from oriH has passed this point [24]. The two strands are therefore single-stranded for different lengths of time. In mammalian genomes, the oriL is located 2/3 apart from the oriH exposing always the same strand as single-stranded. Further, the time of the single-stranded state increases from the oriH to the oriL showing shortest time on oriL, longest time of single-stranded on oriH and long time of single-stranded in the remaining 1/3 part [24]. The faster accumulation of Guanine and Thymine nucleotides in single-stranded DNA then leads to the observed strand-specific AT- and GC-skews [23-27].

However, investigations on mammalian mtDNAs suggest possible alternative L-strand syntheses [33-35] or additional replication modes, which might be tissue- or stage-specific [36-40]. These could substantially reduce the time of single-stranded compared to the conventional strand-displacement mode.

The strand specific accumulations of the Guanine and Thymine nucleotides in the genome of *R. compacta *show typical mutational pattern as proposed by the strand-displacement mode. In particular, the (G + T)/(A + C) ratios in protein-coding sequences shows a gradient along the genome of *R. compata *(Figure 2) in congruence with the conventional strand-displacement mode. The lowest (G + T)/(A + C) ratios are found between the genes *ND5 *and *CYTB*, suggesting that the oriL is located in this region since the shortest time of single-strandedness should cause the lowest bias. The (G + T)/(A + C) ratios increase from *CYTB *to *ND2*, while high ratios are recovered between *ND2 *and *ND5 *(Figure 2). The high ratios suggest that oriH is located close to the end of the *ND2 *gene. Both oriL and oriH have to be expected within genes because large unassigned sequences (UAS) that could contain regulatory elements are missing in the genome of *R. compacta*.

Assuming that there are competing modes of replication for metazoan mitogenomes, the extreme strand specific nucleotide bias in *R. compacta *might indicate that the strand-displacement mode is more dominant than in closely related species. However, the basal protostome clades Platyhelminthes and Chromodorea exhibit a similarly strong strand specific nucleotide bias as *R. compacta *(Figure 1), and similar replication modes might be used in these taxa.

The strand specific mutational bias in *R. compacta *is inverted on the main-coding strand compared to most other deuterostome genomes (Figure 1). This may be explained by an inversion of the direction of replication which has been described for Crinoidea [29], a subgroup of the echinoderms, and several other, unrelated taxa [23]. Only few gene inversions are found in the genome of *R. compacta *compared to genomes of Vertebrata (the *ND6 *and five tRNA genes) or to the enteropneust *Balanoglossus *(the *ND6 *and six tRNA genes) suggesting that the inversion of regulatory elements lead to the inverted replication direction.

### Genetic Code and the tRNA System

All codons are present in the protein-coding genes of *R. compacta*, including the codons TAA and TAG as stop codons. Most assignments of the amino acid codons could be determined on conserved sites within a metazoan alignment using GenDecoder v1.6 [41], whereas five codon assignments could be recovered only with weak support (Table 2). Two of them could be well determined based on weakly conserved or variable sites: UCC (46% Ser) and AAA (69% Lys, no Asn) while only uncertain results were obtained for the codons AUC, UGC, and CUC. However, the codons AUC and UGC present the reverse complement of the anticodon sequence of the tRNA_Ile/GAU _and the tRNA_Cys/GCA_, which support the assignment as Ile and Cys, respectively (Table 2). The CUC codon specifies 27% as Ile, 24% as Phe, and 23% as Leu. Due to the assignment of CUC as Leu in all bilaterian genetic codes deposited in NCBI database, this assignment was also assumed for *R. compacta *(Table 2).

**Table 2:** Codon usage and tRNA anticodons in the pterobranch *R. compacta*.

UUU	554	Phe	UCU	233	Ser	UAU	159	Tyr	UGU	99	Cys
UUC	35	Phe *	UCC	8	Ser	UAC	28	Tyr *	UGC	10	Cys *

UUA	205	Leu *	UCA	32	Ser *	UAA	2	term	UGA	48	Trp *

UUG	210	Leu	UCG	21	Ser	UAG	9	term	UGG	89	Trp

											

CUU	102	Leu	CCU	93	Pro	CAU	70	His	CGU	46	Arg

CUC	5	*Leu?*	CCC	5	Pro	CAC	8	His *	CGC	4	Arg

CUA	19	Leu *	CCA	10	Pro *	CAA	16	Gln *	CGA	15	Arg *

CUG	14	Leu	CCG	11	Pro	CAG	31	Gln	CGG	19	Arg

											

AUU	175	Ile	ACU	76	Thr	AAU	79	Asn	AGU	87	Ser

AUC	9	Ile *	ACC	3	Thr	AAC	8	Asn *	AGC	10	Ser *

AUA	52	Ile	ACA	5	Thr *	AAA	10	Lys	AGA	40	Ser

AUG	96	Met *	ACG	5	Thr	AAG	59	Lys *	AGG	20	Lys

											

GUU	289	Val	GCU	99	Ala	GAU	60	Asp	GGU	158	Gly

GUC	17	Val	GCC	4	Ala	GAC	9	Asp *	GGC	8	Gly

GUA	39	Val *	GCA	12	Ala *	GAA	17	Asp *	GGA	63	Gly *

GUG	64	Val	GCG	11	Ala	GAG	59	Asp	GGG	182	Gly

The total number of codons is shown. The assignment of each codon was detected within a metazoan alignment using GenDecoder server v1.6 [41]. The reverse complements of the anticodons are marked by asterisks, deviations from the versatility rule [15,16,18] are underlined. One codon (question mark) could not be determined with certainty.

The codon usage in the mitochondrial genome of *R. compacta *shows a strong preference of synonymous codons ending with Thymine or Guanine (Figure 1 and Table 2), which is in contrast to most vertebrate mtDNAs [17]. Moreover, the nucleotide composition of the tRNA genes is adapted to the increased GT-content (Table 1), but most anticodons of the tRNA genes still show the typical sequence of other metazoan genomes [19,20] having anticodons GNN for NNY codons, UNN for NNR codons and also UNN for four-fold degenerate codon families (Table 2). Therefore, the reverse complements of the anticodon sequences show usually the low frequent codons (Table 2) rejecting an adaptation of the anticodon sequences to most frequent codons (see [17]). In contrast, this strongly supports that an effective translation system is based on anticodon sequences of tRNA genes with highest versatility to all recognized codons independent of their frequency [15,16,18]. 

Three deviations from the anticodon system with such highest versatility are found in the genome of *R. compacta *(Table 2) which are also recovered in some other deuterostome genomes and which are all correlated to codons with different assignments (Table 3). 

(i) The anticodon sequence for Met is derived as it shows the reverse complement to the AUG codon instead of the AUA codon. This is found in most metazoan genomes and may be important for an effective translation of the start codon AUG (see [17]). The AUA codon shows different assignments within Deuterostomia. In all Echinodermata, the three Enteropneusta and in *R. compacta*, the AUA codon is assigned as Ile (Table 3) while it encodes Met in Chordata (Urochordata, Cephalochordata, and Vertebrata). Since no change in the anticodon of the tRNA_Ile _is found, the additional recognition of the AUA codon by the tRNA_Ile _should be based on other mechanisms which may have arisen in the lineage leading to the Ambulacraria. 

(ii) The tRNA_Ser/GCU _shows also no changes in the anticodon sequences within Deuterostomia and recognises the AGT and AGC (AGY) codons within highest versatility. Due to the missing of a tRNA gene for the codons AGA and AGG (AGR), these codons are partially also recognised by tRNA_Ser/GCU_. The pairing of the anticodon sequence of tRNA_Ser/GCU _to the codon AGA may be possible under special conditions; whereas the recognition of the AGG codon is assumed to be more complicated [15,42]. Within Deuterostomia, only the genomes of Echinodermata show obviously an assignment of Ser for all four codons. The pairing of the AGG codon to the tRNA_Ser/GCU _in echinoderms is explained by a guanosine methylation on the anticodon sequence that enables this pairing [42,43]. The genomes of Chordata and Tunicata possess a different assignment of both codons (AGG and AGA) which is based on an additional tRNA gene for Gly in Tunicata [44] and a unique pathway to recognize these codons as stop codons in Chordata [45,46]. In the genome of *R. compacta*, the codon AGA assigns Ser while the codon AGG is reassigned to Lys (Table 3). 

(iii) The assignment of the codon AGG as Lys is supported for the genome of *Rhabdopleura *by eight highly conserved sites (83% with Lys) and two weakly conserved sites (63% with Lys), but has not been detected in other deuterostome mtDNAs. However, several taxa within Arthropoda also have an assignment of AGG for Lys. All these genomes and the genome of *R. compacta *present an unusual anticodon sequence of tRNA_Lys _showing the sequence "CUU" instead of "UUU" (Table 3) [47]. Only the second codon position of the codon AGG mismatches to the anticodon sequence tRNA_Lys/CUU _and may enable the codon reassignment to Lys. Notably, the arthropod lineage shows also mtDNAs with tRNA_Lys/CUU _or tRNA_Lys/UUU _but missing the reassignment. The distributions of the anticodon sequences within arthropods suggest an ancestral mutation in the tRNA_Lys _but could not support (but also not reject) an ancestral assignment of AGG as Lys [47]. Therefore, several independent back-mutations to the usual anticodon sequence "UUU" and several reassignments of AGG to Lys may have occurred [47]. Similar to this, the genomes of Echinodermata and the enteropneust *Balanoglossus *spp. have also the derive anticodon sequence "CUU" while the genome of the enteropneust *Saccoglossus *shows the usual anticodon sequence of "UUU" (Table 3). All these genomes lack the reassignment of AGG to Lys. Furthermore, the translation efficiency for the codon AAA by the tRNA_Lys/CUU _could be reduced due to deviation from the highest versatility in the 3^rd ^codon site (anticodon sequence changed to "C" instead of "U" in the first site) and may have caused the lack of the AAA codons in the genomes of *Balanoglossus *[30] as well as the reassignment of the codon AAA to Asn in echinoderms [48]. The recognition of the codon AAA by the tRNA_Asn/GUU _in echinoderm genomes may be based on a further mutation on position 33 in the anticodon loop of the tRNA_Asn _[31,48] which is missing in pterobranch and enteropneust genomes (Table 3).

**Table 3:** Mitochondrial Genetic Code of Deuterostomia and selected Protostomia.

	Codon AUA	Codon AAA	Codon AGG	Codon AGA	Anticodon loop of tRNA_Asn_	Anticodon loop of tRNA_Lys_	Source/CodTab
*Rhabdopleura compacta*	Ile	*Lys*	Lys	Ser	UU GUU AC	CU **C**UU AA	this study, table 1

*Balanoglossus spp*.	Ile	-	-	Ser	CU GUU AA	CU **C**UU AA	[30,31]

*Saccoglossus kowalevskii*	Ile	Lys	-	Ser	CU GUU AA	CU UUU AA	NC_007438

Echinodermata	Ile	Asn	Ser	Ser	C**C **GUU AA	CU **C**UU AA	CodTab 9

Tunicata	Met	Lys	Gly	Gly	CU GUU AA	CU UUU AA	CodTab13

Vertebrata	Met	Lys	term	term	CU GUU AA	CU UUU AA	CodTab 2

Cephalochordata	Met	Lys	-	Ser	CU GUU AA	CU UUU AA	CodTab 5

invertebrates	Met	Lys	Ser	Ser		CU UUU AA	CodTab 5

hypothetical ancestral state in Arthropoda	Met	Lys	Lys/Ser	Ser		CU **C**UU AA	[47]

The variable codon assignments within mtDNA of Deuterostomia and selected anticodon loop sequences (the anticodon sequences are underlined) are shown. The entire genetic codes available at the web site http://www.ncbi.nlm.nih.gov/Taxonomy/Utils/wprintgc.cgi are given by the codon table number (CodTab). Amino acids are shown in the 3-letters-abbreviations. Low frequent codons are given by cursive amino acid abbreviations while unpredictable or completely missing codons are shown by dash.

In summary, the distribution of the tRNA_Lys _anticodon sequence within Deuterostomia and the reassignments of the codon AAA (in Echinodermata) and AGG (*R. compacta*) suggest a tRNA_Lys/CUU _as an apomorphic character for the taxon Ambulacraria (Table 3). Furthermore, our data support the codon reassignments as a two-step-process: The first step was the reduced recognition in mtDNAs of Ambulacraria as a consequence of a mutation in tRNA_Lys/CUU _abandoning AAA and the lack of a suitable tRNA for codon AGG. These reductions paved the way for adaptations in two other tRNAs to recognize these codons: AGG by tRNA_Lys/CUU _in *R. compacta *and AAA by tRNA_Asn/GUU _in Echinodermata. Both steps were forced by an effective translation system in congruence to the "Modified Ambiguous Intermediate Theory of Codon Reassignment" [49-51]. The alternative "Codon Capture Hypothesis of Codon Reassignment" [52] assumes mutational pressure in the first step leading to codon losses in the genome. This seems to be unlikely because the genome of *R. compacta *presents a strong mutational bias in all protein-coding genes, but no codon is missing.

### Protein-Coding Genes

**Figure 3:**
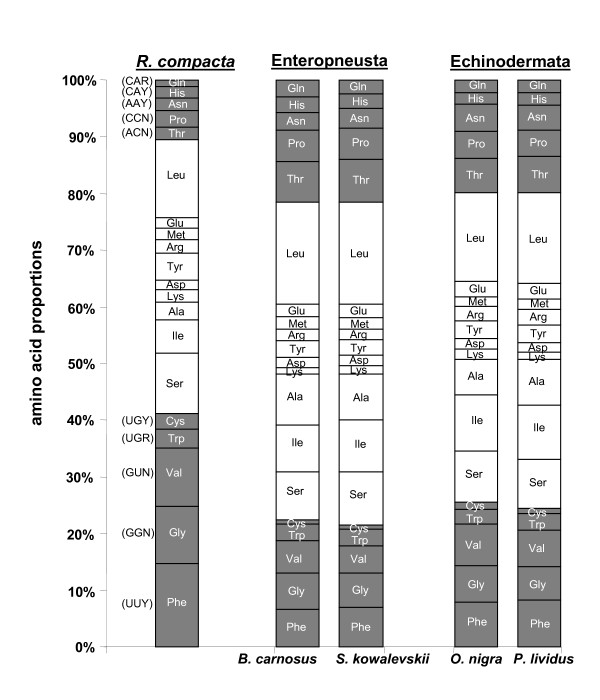
**Amino acid composition of the 13 protein-coding genes in *Rhabdopleura compacta *compared to closely related deuterostome species: the enteropneusts *Balanoglossus carnosus *and *Saccoglossus kowalevskii*, the sea urchin *Paracentrotus lividus *and the brittle star *Ophiocomina nigra***. Amino acids solely encoded by GT or AC rich codons are indicated by white letters. These codons are listed on the left and are identical for all mtDNAs of Deuterostomia.

The GT nucleotide composition has an important influence on the protein composition of nuclear genomes [53]. Similarly, the mutational bias in the genome of *R. compacta *strongly influences the protein sequences, even if highest GT-bias is detected in the third codon position (Figure 1). The GT-rich main-coding strand of *R. compacta *exhibits all protein-coding genes showing an increase of GT-rich codons at the expense of AC-rich codons. This leads to unusual amino acid composition in all proteins (Figure 3). The abundance is increased for Phe, Gly, Val, Trp which all are encoded by exclusively GT-rich codons (Figure 3). In contrast, the amino acids Thr, Pro, Asn, His, and Gln are decoded solely by AC-rich codons and are decreased in their abundances in the proteins of *R. compacta *compared to the proteins of closely related species (Figure 3).

**Figure 4:**
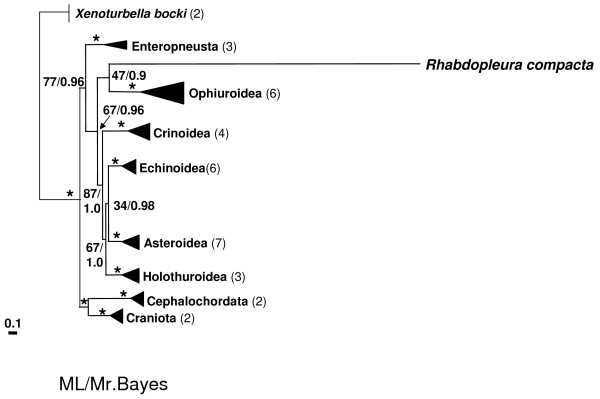
**Maximum likelihood tree of the major deuterostome groups excluding tunicates using the amino acid sequences of all thirteen mitochondrial protein-coding genes**. The numbers on the nodes show the bootstrap percentages (BP) for ML and the posterior probabilities (PP) of the Bayesian analyses in this order. Asterisks indicate highest support values. The numbers in parenthesis behind the deuterostome groups show the number of analyzed species. The length of the triangles for each group indicates the variability within the group. The extreme long branch leading to the pterobranch *R. compacta *is based on its highly different protein-composition compared to other deuterostome sequences (for details see text).

This strong bias within the amino acid sequence hampered the phylogenetic analyses of the protein-coding genes leading to extreme long branch lengths (Figure 4). Both ML and the Bayesian approaches resulted in a meaningless sistergroup relationship of *R. compacta *to the echinoderm subgroup Ophiuroidea, hence rejecting a monophyletic Echinodermata and Hemichordata (Figure 4). While the monophyly of Echinodermata is well supported by several other analyses [6], the mitochondrial genomes of Ophiuroidea show an accelerated evolutionary rate [11,54,55]. Thus, the grouping of *R. compacta *and Ophiuroidea most likely is caused by a long branch artefact due to the unusual protein composition. However, the protein composition of *R. compacta *suggests a long time of adaptive evolution under the same mutational pressure.

### Gene Order Evolution

The mtDNAs of most deuterostome groups show similar gene arrangements, especially the order of the protein-coding genes. The consensus gene order of vertebrate genomes is assumed to represent the ancestral arrangement for Deuterostomia, if not for Metazoa [8]. Only the genomes of Tunicata show unique gene arrangements (including two additionally tRNA genes) featuring all genes on one strand and having unusual high rearrangement rates within the group (e.g. [13]). The mitochondrial genome of *R. compacta *presents a new gene arrangement showing high differences in the tRNA and rRNA gene order compared to the assumed ancestral deuterostome as well as to the known hemichordate arrangements (Additional file 1, Figure S1).

**Figure 5:**
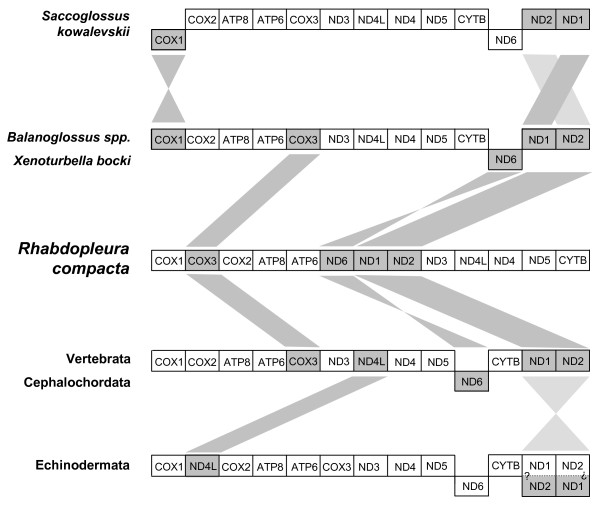
**Protein-coding gene order comparison of the mtDNAs from the pterobranch *Rhabdopleura compacta *to the enteropneusts, *Xenoturbella bocki*, the ground pattern of echinoderms and the consensus arrangement of vertebrates**. Genes located above the middle line are transcribed from the heavy strand whereas those located below the middle line are transcribed from the light strand. The question marks indicate the unknown ancestral strand affiliation of the fragment containing the *ND1 *and *ND2 *genes in echinoderm genomes (see [11]). Rearrangement steps between the gene orders are marked by grey straight lines (transpositions) and grey crossed lines (inversions).

With regard to the order of the protein-coding genes the arrangement of *Balanoglossus *(identical to *Xenturbella bocki*) appears to represent the ancestral state within Hemichordata, since only one translocation of *CYTB *or *ND6 *from the vertebrate/cephalochordate arrangement has to be hypothesized (Figure 5).

Within Hemichordata, two rearrangements are necessary to interconvert the basal *Balanoglossus *gene order into that of *Saccoglossus kovalewskii *(the inversion of *COX1*, and the transposition of either *ND1 *or *ND2*). The gene order of *R. compacta *needs three steps to be derived from the ancestral *Balanoglossus *order (inversion of *ND6*, translocation of *COX3*, and transposition of the fragment containing *ND1 *and *ND2*) (Figure 5). However, these rearrangements have to be addressed as autapomorphies and thus, no synapomorphic characters could be detected in gene order evolution within Hemichordata, which would allow phylogenetic reconstruction within the group.

Notably, the gene order of *R. compacta *can be derived directly from the assumed basal deuterostome arrangement (Vertebrata) by the same number of three rearrangements, which however would render Hemichordata paraphyletic (Figure 5).

Although the ancestral arrangement of the fragment containing *ND1 *and *ND2 *cannot be surely determined [11], the echinoderm genomes show an apomorphic translocation of the *ND4L *gene, which is not recovered in any other deuterostome genome (Figure 5).

## Conclusion

The unusual mitochondrial genome of *R. compacta *features a strong, reversed asymmetrical mutational constraint compared to most other deuterostome genomes. This may have arisen by an inversion of the replication direction in the lineage leading to *R. compacta *and by an adaptation to this bias in the protein sequences. Due to this bias, phylogenetic analyses of protein-coding genes failed. However, the tRNA system supports the monophyly of Ambulacraria, comprising Hemichordata and Echinodermata by the synapomorphic characters tRNA_Lys/CUU _and the assignment of ATA as Ile. The strong strand-specific mutational bias in the genome of *R. compacta *allows further important insights in the evolution of the tRNA system. The observed codon usage and their anticodon sequences strongly support a selection pressure to an efficient translation system possessing anticodon sequences which pair most easily with all recognized codons, independent of their frequency (anticodons with highest versatility) similar to most tRNA genes of fungal mtDNAs [15]. Mutations in the anticodon sequence changing the recognition efficiency could lead to a bias in the codon distributions and could cause the deletion of codons from the genome. Furthermore, similar mutations in the anticodon sequence could cause similar adaption processes in the tRNA translation system as found in the convergent assignment of AGG as Lys in the genome of *R. compacta *and some arthropod genomes. Our analyses support the disappearance and reassignment of codons in response to a selection pressure for an efficient translation system as described in the "Modified Ambiguous Intermediate Theory of Codon Reassignment" [49-51].

## Methods

### DNA Extraction, Amplification, Sequencing and Annotation

About 50 adult zooids of *Rhabdopleura compacta *Hincks, 1880 were dissected from their coenecia, which were attached to disarticulated shells of *Glycymeris glycymeris*, dredged off from Stoke Point (near Plymouth, UK, 50°17'N, 4°01'W) in Mai 2006 from a depth of 21-24 m. Genomic DNA was extracted using phenol - chloroform extraction, following proteinase K digestion. The complete mitochondrial genome was amplified using two overlapping fragments. The first fragment was amplified with the specific primer pair GTGGTGGAGTACCCTTTTAAGACTG and GACCCAATAGTTGAAGCATGATGCC, which was determined from the *COX1 *sequences that we found in our *Rhabdopleura *EST (M. Perseke et al., unpublished). The second fragment was amplified with the specific primer pair GTTTACTTTGGGGGGTTGCACTGG and CCAACGCTCAATCAGCTTCAAGAGC, which was designed based on the first *COX1 *fragment sequenced. The PCR reactions were performed with a Mastercycler machine (Eppendorf AG, Hamburg, Germany). The cycling was set up with an initial denaturing step at 94°C for 3 minutes, followed by 35 cycles of denaturing at 94°C for 60 seconds, annealing at 60°C for 30 seconds and elongation at 68°C for 5 minutes.

The 25 μl PCR reaction amounted 0.3U of the Phusion High-Fidelity DNA polymerase (Finnzymes, Espoo, Finland) and 0.3 ng DNA as well as 5 μl 5× Phusion GC buffer (Finnzymes), 2.5 μl dNTP mix (10 mM each) and 1 μl of forward and reverse primers, respectively (10 μM each). The PCR product of the large fragment was sequenced by primer walking as described by Perseke et al. [54]. Additional starting points for faster primer walking were obtained by preparing a "mini" DNA library using four-cutter restriction enzymes and the pGem-T plasmid Vector (Promega). All PCR products were sequenced directly on an ABI 3100 automated sequencer (Applied Biosystems) using the BigDye Termination v3.1 Cycle Sequencing Kit (Applied Biosystems). After concatenating the sequences to the complete genome, all protein-coding genes and the rRNA genes were identified by alignments. All tRNA genes were identified by using the tRNAscan-SE 1.23 server [56]. The entire mitochondrial DNA sequence was submitted to the GenBank under the accession number FN908482.

### Analysis of Nucleotide Composition and the Genetic Code

The strand biases of the main-coding strand, given as the AT-skew [(A-T)/(A + T)] and the GC-skew [(G-C)/(G + C)] [57], were calculated using an automatic tool developed for this purpose for both the complete genome of *R. compacta *and all complete mitogenomes available in NCBI RefSeq 41 [58]. In addition, the AT- and GC-skews were computed separately excluding protein-coding sequences and further using only the 3^rd ^codon positions of the protein-coding genes of *R. compacta*.

The values of the (T + G)/(C + A) ratios were determined for each position in the genome of *R. compacta *using a window starting 149 positions to the left and ending 150 positions to the right.

The genetic code of the *R. compacta *genome was predicted by evaluating four different degrees of conserved codons (highly conserved, conserved, weakly conserved and variable sites) in the alignment of 56 metazoan sequences implemented in the GenDecoder server v1.6 [41].

### Analysis of Gene Order

The genome rearrangements were studied using the web-based CREx software [59]. The gene order of *R. compacta *was compared to the following deuterostome genomes: both *Balanoglossus *species [11,30]; *Saccoglossus kowalevskii *(NC_007438), *Xenoturbella bocki *[10,14], the putative ancestral arrangement in Echinodermata [11], the consensus gene order of Vertebrata [12], the ancestral order of Cephalochordata [9] and all known gene orders of Tunicata [13]. Further, the gene order was compared to Platyhelminthes [60], and Nematoda [61]. All comparisons were performed twice - with and without - the tRNA and rRNA genes.

### Phylogenetic Analyses of Protein-Coding Sequences

The phylogenetic analyses of the protein-coding genes were carried out by adding the *R. compacta *protein sequences to the taxon set "Deuterostomia" from Perseke et al. [11]. All protein-coding genes were aligned separately using the T-Coffee server with the default parameter set [62]. The sequences were then truncated, so that no genes started and ended with a gap. Finally, they were concatenated. The "mtRev" model of amino acids was selected and the gamma shape parameter and the proportion of invariable sites were determined using PhyML v.2.4.4 [63]. The Maximum Likelihood (ML) analyses were carried out with PhyML v.2.4.4 [63] using the determined parameters and six categories of substitution rates. The robustness of bifurcations was estimated with bootstrap analyses (100 replicates). The Bayesian analyses were performed with MrBayes v.3.1.2 [64] using the determined parameters and six categories of substitution rates. The calculation was run for 1,000,000 generations, with a sampling frequency of 10 generations and burn-in of the first 25,000 trees. The remaining trees were tested for stability of the likelihood values and used to compute the posterior probabilities.

## Authors' contributions

This study was carried out in collaboration between the working groups of MS and PFS. DB, MS and PFS defined the research theme. MP, MS and DB designed methods and experiments. MP collected specimens and extracted DNA. MP and JH amplified, sequenced and analyzed the mt genome. MB calculated and displayed the nucleotide biases. MP interpreted the results and PFS, MS and DB discussed the analyses, interpretation, and presentation. All authors have contributed to the manuscript and approved the submitted version.

## Supplementary Material

Additional file 1 **Figure S1 -- Gene order comparison of the mtDNAs from the pterobranch *Rhabdopleura compacta *to the enteropneust genomes**. Genes located above the middle line are transcribed from the heavy strand whereas those located below the middle line are transcribed from the light strand. Grey regions highlight conserved protein-coding gene arrangements. The black lines show the tRNA rearrangements (transposition, inversion and reverse transposition) within the conserved blocks. Click here for file
