# Supplementary figures and images for: The enigmatic mitochondrial genome of Rhabdopleura compacta (Pterobranchia) reveals insights into selection of an efficient tRNA system and supports monophyly of Ambulacraria
Source: BMC Evol Biol. 2011 May 20;11:134. doi: 10.1186/1471-2148-11-134 (PMC3121625; doi:10.1186/1471-2148-11-134)

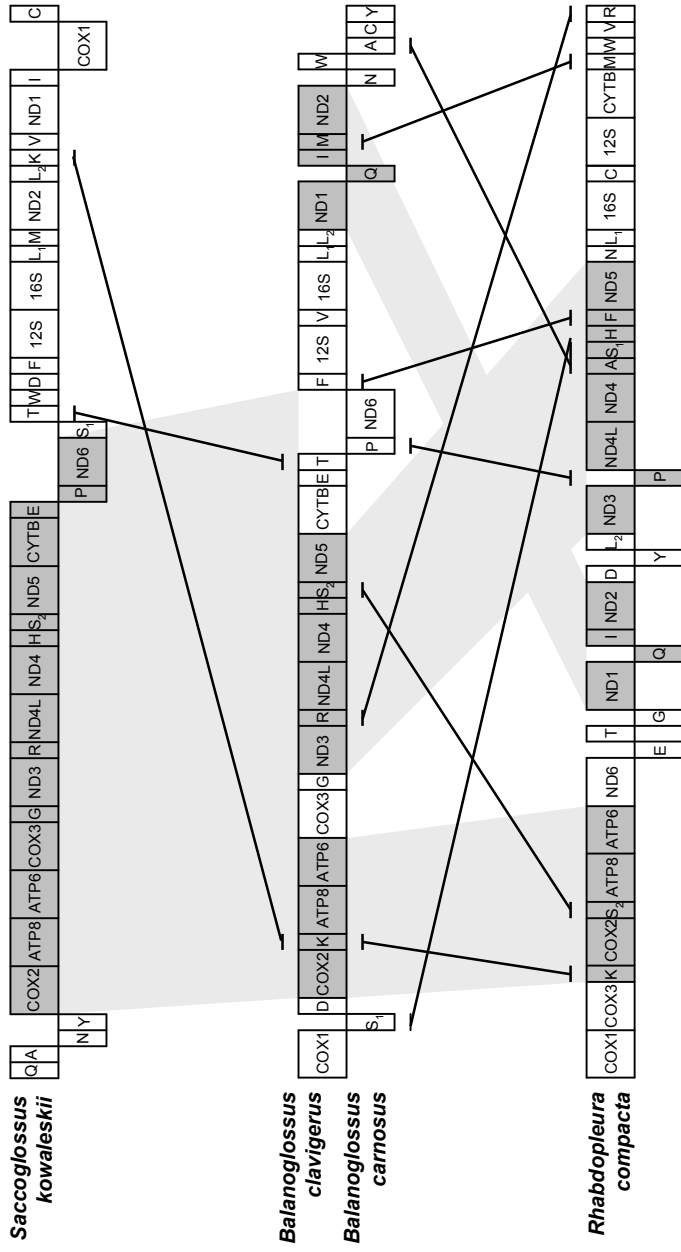

Supplement: Additional file 1 — Figure S1 -- Gene order comparison of the mtDNAs from the pterobranch Rhabdopleura compacta to the enteropneust genomes. Genes located above the middle line are transcribed from the heavy strand whereas those located below the middle line are transcribed from the light strand. Grey regions highlight conserved protein-coding gene arrangements. The black lines show the tRNA rearrangements (transposition, inversion and reverse transposition) within the conserved blocks. [file 1471-2148-11-134-S1.PDF]
